# The Philosophy of Exudative Inflammation

**Published:** 1898-02

**Authors:** John Rogers

**Affiliations:** New York


### International Dental Journal.

Vol. XIX. February, 1898. No. 2.

### Original Communications.[1]

### THE PHILOSOPHY OF EXUDATIVE INFLAMMATION.[2]

BY JOHN ROGERS, M.D., NEW YORK.

The reaction which follows a traumatism or the introduction into the tissues of micro-organisms is conveniently called an exudative inflammation. It is a term which expresses the difference between the more violent reaction, which is accompanied by redness, heat, pain, and swelling, and the less violent and in general more chronic response produced by other forms of injury. For injury of one character or another is now conceded to be the origin of such changes as cirrhosis of the liver, endarteritis, the degenerations of phosphorus-poisoning, the manifestations of syphilis, new growths like sarcoma, etc.

The exudative form of the process is the most common. It occurs not only after the lodgement in the tissues of micro-organisms, but also after every traumatism. It is nature's response to an injury, and the changes which take place are of a conservative and reparative character. To show this it is necessary to examine each stage or step in such a reaction. In its simplest forms the exu-

[1] The editor and publishers are not responsible for the views of authors of papers published in this department, nor for any claim to novelty, or otherwise, that may be made by them. No papers will be received for this department that have appeared in any other journal published in the country.

[2] Read before The New York Institute of Stomatology, Tuesday evening, November 2, 1897.

dative part of the process is not so prominent as the part which is reparative. If, for example, a simple incised wound is made, aseptically, in the skin of a rabbit, the reaction is manifestly reparative. A true exudative inflammation follows, but the exudate is slight. Plasma, white and red cells, escape from the divided vessels. Fibrin rapidly forms and glues together the severed edges of the wound. The neighboring vessels dilate, and there is some emigration of leucocytes, which absorb the fragments of inert *débris* resulting from the few fixed cells killed by the stroke of the knife. Incidentally it is worthy of note that a cell does not perish unless its nucleus is damaged.

This incorporation in the white cells of living or inert particles will be discussed later, but is mentioned to show the correlation of all the different phenomena of an exudative inflammation.

At the same time with this clearing of the field karyokinetic changes are observable in the neighboring fixed cells. Out of one cell two are formed. The nucleus first shows striations, which collect at opposite sides, and around each half thus made protoplasm is heaped up and the cell divides. The result is an epithelioid cell or fibroblast; it is formative, rounded or irregular in shape, and larger than a leucocyte. The fibroblasts gradually elongate and shrink, and processes develop; in short, new connective tissue develops from the pre-existing fixed cells and the parts are restored to their original condition. The important part in this reaction is the proliferation of the fixed cells. It is brought about by indirect or direct division, by mitosis or amitosis, as it is technically called. By mitosis is meant division preceded by certain changes within the nucleus which are accompanied by the formation of peculiar lines and figures. By amitosis, or direct division, is meant the splitting of the cell and its nucleus into two without any preliminary alterations. In direct division the cell is not supposed to be as vigorous as in indirect division.

If we examine the phenomena following a more extensive aseptic wound the exudate is found to occupy a more prominent position. An example is furnished by snipping off a piece of the surface of a frog's tongue. Under the microscope there is seen to be an initial acceleration followed by a slowing of the blood-current; at the same time the vessels become dilated and congested.

Thoma has demonstrated that the well-known marginal position of the leucocytes which ensues is due to the difference in specific gravity of the red and white cells. Also that the emigration of the leucocytes takes place in the line of junction of the endothelial cells of the capillaries and small veins. Diapedesis of the red cells occurs at the same points, as does also the escape of most of the plasma. The latter forms a coagulum over the surface of the wound and occludes the divided vessels. Then follows the repair of the injury by granulation. The pre-existing fixed connective-tissue cells and the endothelial cells of the capillaries proliferate. From the connective-tissue cells are produced epithelioid cells or fibroblasts; these are amœboid, and with difficulty to be distinguished from the leucocytes which have emigrated from the now congested vessels. The fibroblasts are embryonic in character and actively multiply; they collect in small heaps, into which a prolongation of an endothelial cell of an adjoining capillary is sooner or later pushed. This solid prolongation may join with others from its own or another vessel. Eventually it becomes hollow, and blood circulates through it. Meanwhile, the wandering cells, which are derived mainly from the leucocytes of the blood, and probably to a less extent from the proliferation of the pre-existing, fixed, connective-tissue and endothelial cells, cover the surface of the wound and infiltrate the coagulum formed just after the reception of the injury. The numbers of the wandering cells correspond directly with the amount of irritation and consequent destruction to which the surface of the wound is subjected. The same thing can be said of the exudation of plasma. If an ill-fitting shoe is constantly rubbing an ulcer of the heel, pus is present in abundance. Remove the shoe and stop the injury to the growing cells and the pus vanishes.

The purpose of the wandering cells is to act as scavengers; they incorporate and digest or carry off detritus, and the greater the emigration the more permeable the vessels become and the greater is the escape of plasma. Some of the scavengers disintegrate and are absorbed by others; many are cast off as part of the pus; a smaller quantity pass into the lymphatic system. A large number of the wandering cells serve as food to the growing fibroblasts. They probably do not, however, change into fixed cells, although the latter do become wandering cells. The amount of plasma, as previously remarked, corresponds with the cellular activity. In an aseptic wound it serves a flushing out and a nutritive purpose. It aids in the removal of effete matter and supplies nourishment to the rapidly growing cells.

My object in thus hastily reviewing the most prominent features in healing by primary union and by granulation is to emphasize the fact that in these processes there occurs an exudative inflammation, which is anatomically the same as that which follows bacterial infection. That, in short, every manifestation of what we call inflammation, every term to which the ending of "itis" is added and many to which it is not, signifies that the body, made up of many elements, defends itself by assigning to each element a definite duty, which each performs according to need. The marshalling of all the forces may only be evident when micro-organisms are to be combated. All are constantly in line and ready to repel attack, but only one or two companies of the army may need to be called into action for a minor injury like an aseptic wound or contusion. For a thorough understanding of what is meant by inflammation it is necessary to carry in mind a comprehensive view of the whole field.

We pass now to the more violent form of exudative inflammation, that which follows the lodgement and growth within the tissues of micro-organisms. In this the white cells are so numerous and the *débris* so abundant that the inflammatory reaction is more commonly designated as suppurative. But it differs anatomically only in degree, not in kind, from the reaction following a simple aseptic traumatism, and its purpose is the same,—namely, the repair of the injury.

If a little of a pure culture of the staphylococcus pyogenes aureus is inoculated under the skin of a rabbit a circumscribed abscess develops. In and around the point of inoculation there is a degeneration and death of cells coincident with a multiplication of the bacteria. The surrounding vessels are congested and there is an emigration of leucocytes, some of which are mono- and others polynucleated. There is also an exudation of plasma and some diapedesis of red cells. A little later many of the bacteria can be made out in the interior of the emigrated white cells and in the endothelial cells of the neighboring capillaries. This incorporation of bacteria in living cells will presently be described as the process of phagocytosis. The multiplication of the bacteria gradually ceases, then the central portion of the disturbance becomes pus, or a mass of broken-down *débris*, bacteria, fatty matter, white cells, etc., and is discharged externally, leaving a cavity to be healed by granulation. An example of a less favorable termination is furnished by the inoculation of a chicken with the vibrio of Metschnikoff. In this instance the bacteria do not remain localized, but spread from the point of infection almost without hinderance into the blood-vessels and lymphatics and the general circulation. There is extensive degeneration of all the cells near which the bacteria appear. This includes the cells which constitute the walls of the vessels, and with the destruction of the walls of the vessels hemorrhage occurs. At the same time in the region of the bacteria there is a copious exudation of a sero-sanguinolent fluid; but there is no emigration of leucocytes and no phagocytosis, which are two significant points of difference between the localized abscess and the general infection. In the former the leucocytes are numerous and close around the bacteria; in the case of general infection there is no massing of leucocytes; the few which appear are evidently degenerated and quickly perish.

To be complete, croupous, necrotic, or diphtheritic inflammation should be mentioned. But it mainly differs from the localized abscess and the diffuse cellutitis with general sepsis in that a mass of cells perish more or less rapidly, and together instead of slowly and one after another. Furthermore, in croupous inflammation the dead mass does not disintegrate and liquefy till after the lapse of some little time. But the philosophy of the minute phenomena is practically the same, and it is to this I want to call your attention.

The first elements to be considered are the wandering cells. Many are derived from the proliferated fixed tissue-cells, but the larger proportion have been proved by Cohnheim to be the emigrated leucocytes of the blood. These differ in their appearance, size, probable ultimate origin, and staining reactions. One of the latest classifications of human leucocytes enumerates six distinct kinds. Of these, at least two have been ascertained to have definite functions.

Their presence or absence around an injurious body is dependent upon the latter's attractive or repulsive powers, upon a positive or negative chemiotaxis, as it is called. This simple fact was not grasped till attention was called to the approach in water of a certain fungus towards oak-bark, and its retreat from a substance like sodium chloride. Then other low forms of life, including the unicellular amœba, were found to possess the same property of voluntary movement in approaching their prey or leaving a dangerous neighborhood. Observation speedily extended to the leucocytes or unicellular organisms in mammals, and their behavior in this respect towards bacteria.

If a capillary tube containing in its centre certain microorganisms is planted under the skin of a rabbit it will speedily become filled with leucocytes, and in this instance the bacteria have positive chemiotactic properties. Other species of bacteria similarly implanted show negative chemiotactic properties,—they repel the white cells from their neighborhood, and within certain limits this indicates that the animal in which this property is manifested will be overcome by the growth of the bacteria. But as it was observed that the fungus which was at first repelled by sodium chloride can in time, by deprivation of water, be made to approach this substance, so, as regards bacteria and the emigrated leucocytes, can a negative be made to change into a positive chemiotaxis. Recent study has somewhat modified this view, in that now it is known that all bacteria exhibit, at the outset of their parasitic existence, a greater or less amount of negative chemiotaxis. The more speedily this changes to positive the better for the host.

Let me give an example of this change. It has been stated that the vibrio of Metschnikoff repels the leucocytes of a chicken into which it has been introduced, and that this is followed by the death of the chicken. If, however, the chicken is previously rendered immune to this vibrio and is then inoculated with a virulent culture, the usual negative chemiotaxis is found to have become decidedly positive. The chicken which would ordinarily die survives, and indeed manifests but slight evidence of disturbance.

Metschnikoff was the first to show that the leucocytes which exhibit this positive chemiotaxis towards bacteria subsequently ingest and destroy them; and he believed that this destructive power of its leucocytes alone determined the survival of the animal. If the leucocytes were repelled from the bacteria and so could not absorb them the animal perished. The process is much more complex, as will be presently demonstrated. Comparative pathology has supplied most of the light on this subject.

The amœba—a unicellular organism comparable to the leucocyte—approaches and ingests certain living organisms or diatoms which serve as its food. In the amœba the diatom lies in a cavity containing an acid fluid, and by this is gradually disintegrated. But if certain other forms of minute life are taken up by the amœba the digestive fluid in the vacuole is *not* acid, and the absorbed micro-organism does not perish, but grows and kills its host. There are found in the bodies of the entire animal kingdom analogous, unicellular, amœboid organisms which originate in and belong to the tissues, named from their derivation mesodermal, and the discovery of analogous properties in these cells throughout the different animal species is the proof of what may sometimes be surmised but not always demonstrated in man.

Investigation has shown that the same laws prevail throughout the whole animal kingdom. Even the very lowest forms of life, in which there is no nervous system and no closed vascular system, are afflicted with parasites, which are approached, ingested, and thus destroyed by the wandering, mesodermal, amœboid cells or leucocytes. If the parasites are not thus destroyed, the host perishes. A little higher in the animal scale the leucocytes begin to appear to be of more than one variety; they have different staining properties and different functions; some are phagocytic, that is, have the power of ingesting parasites, others are not so. In the mammalia the differentiation, as might be expected, becomes most marked.

Man is known to be provided with two or at most three classes of leucocytes, which, as they appear to have distinct duties, require some description. The other varieties are as yet mainly of scientific interest and need only be mentioned.

The hyaline leucocyte is phagocytic, exists most abundantly in and near the serous membranes, and has a single nucleus, which stains with basic, aniline dyes. The *finely* granular oxyphile cell is the ordinary pus-cell; it is oxyphilous, or stains with acid dyes, and is also phagocytic. The *coarsely* granular oxyphile cell, or the eosinophile leucocyte, stains with acid dyes, is polynucleated but not phagocytic. Its properties are not, strictly speaking, proved, but are inferred from what is known about closely analogous cells in lower animals.

It is necessary now to return for a moment to the theories of Metschnikoff. The discoverer of phagocytosis believed that this process alone controlled recovery after bacterial infection. That it is of great importance is shown by a simple experiment of much practical applicability. The guinea-pig is immune to inoculation with the spores of tetanus bacilli. Ordinarily the leucocytes approach and absorb them, and the animal recovers with but slight symptoms of disturbance. But if the spores are first encased in paper and then implanted in the pig, the leucocytes cannot come in contact with the spores, and the guinea-pig dies. A similar course of events can be supposed after infection of a mass of devitalized human tissue. The dead matter acts like a fence enclosing the bacteria. It prevents the latter from being reached by the wandering cells.

It is well known that debilitated persons are an easy prey to every kind of infection, and some of the earliest experiments clearly indicate that the general vitality of the individual is an important factor in phagocytosis. The chicken, for example, is immune to anthrax. The *spores* of this bacillus are taken up by the leucocytes, but seem very resistant; they are difficult of digestion, and they survive in the leucocytes a considerable time. If now the bird is immersed in cold water and its strength thus impaired, the spores begin to grow and destroy the phagocytes; eventually they kill the chicken.

The white rat is similarly immune to anthrax. But if the rat is first made to turn a wheel until exhausted and is then inoculated, the result is the same in all respects as in the exhausted chicken,—death.

It was long doubted whether phagocytosis of living bacteria ever occurred; but now there is no question but that it does. It has been proved by numberless experiments; but the estimation of its importance has not been so easily settled.

One of the principal reasons for questioning the efficacy of phagocytosis as Metschnikoff understood it, was the fact that bacteria were often found to be dead before any leucocytes approached them. Then it was discovered that simple defibrinated blood had decided germicidal properties. The shed serum was actually more fatal to bacteria in many instances than that found in living tissues. Further experimentation demonstrated that the antiseptic substance of the serum was associated with the leucocytes; for an exudate rich in leucocytes was more bactericidal than the serum obtained by defibrination. This led to a study of these cells, and in the higher animals, as mentioned before, several distinct classes of leucocytes were identified. Kanthack and Hardy led the way in elucidating their functions. The frog is immune to anthrax. They found that anthrax bacilli suspended in frog's lymph were approached by leucocytes containing granules which stained with eosin. These granules were discharged and the bacilli apparently paralyzed or killed. The eosinophilous cells then withdrew, and were succeeded by mononucleated cells staining with methylene blue. These acted as phagocytes.

Thus something which had to do with the fluid part of the blood and tissues, or at least with the unformed elements, usually affects the bacteria before phagocytosis can occur. A well-known example of the power of this mysterious something is furnished by the clumping and loss of motility of typhoid bacilli in Widal's test for typhoid fever. The cell-free serum of an individual suffering from this disease, when mixed with a culture of actively moving typhoid bacilli, causes the latter within a few moments to cluster together in a mass, to lose their motility, and to perish.

All the antidotal serums are obtained from the blood of immune animals, and are now known to contain two substances, one of which is bactericidal alone, the other both bactericidal and antitoxic; that is, it destroys the bacteria and in addition neutralizes their products. These substances are of complicated composition, and have been compared by Behring to the electricity in a magnet. It seems impossible to isolate or measure them. They are not analogous to a simple antiseptic, for if they were, the more of an immunizing serum which could be administered to a diseased animal the greater ought to be that animal's resisting power. Such is not the case. Neither do these bactericidal and antitoxic substances neutralize bacteria and their poisons like an acid does an alkali; for if a guinea-pig and a mouse are simultaneously injected each with an equal amount of immunizing serum and tetanus toxin, the larger animal will die while the smaller will survive.

The immunizing body or bodies, as there are more than one, have been given the name of alexins. They are probably derived from the nuclein of the wandering and fixed tissue cells. By injecting a vegetable irritant into the pleural cavity of guinea-pigs Büchner obtained a germ-free, purulent exudate, which was more bactericidal than normal blood. Kossel has proved that when the nuclein is separated the residue of the exudate loses much of its bactericidal power, but the solution of the separated nuclein still possesses it in an active form.

In this connection it is well to mention the investigations of Hankin. Following the lead of Kanthack and Hardy, he claims to have seen the granules in the eosinophilous cells of a purulent exudate collect on one side of these cells and then disappear. Coincidentally with this disappearance of granules from the cells the bactericidal power of the serum increased. Enough has now been established to prove that at least some of the cells produce in the presence of bacteria alexins or defensive proteids, which affect injuriously the bacteria before or in conjunction with the phagocytes.

As long ago as 1890, Tizzoni and Cattani stated that these defensive proteids were of the nature of an enzyme, or digestive ferment. The diatom contained in the vacuole within the amœba which devours it is destroyed by an acid fluid in this vacuole. The anthrax bacillus is similarly seen to disintegrate within a vacuole in a leucocyte of frog's lymph.

If attenuated anthrax bacilli are implanted in a glass tube under a rabbit's skin, many are found to be dead before phagocytosis has occurred. But both those within and those without the cells, dead and disintegrating in both instances, present the same general characteristics and reactions, and it is reasonable to suppose that the same agent destroys both the intra- and the extracellular organism. That this is a ferment seems to have been proved by Leber. He excited an aseptic, purulent inflammation by inserting a sterilized copper plate in the anterior chamber of a rabbit's eye. This exudate freed of cells was then found to be capable of digesting proteid material.

Another rather noticeable analogy of the alexins with the digestive secretions of the body is furnished by the reaction of the blood in the course of an infection. Many experiments show that an increase in its alkalinity favors immunity, and Calabrese has demonstrated, by inoculation of susceptible animals with cultures of gradually increasing strength, that the alkalinity of the blood increased with each inoculation, and that it reached its maximun when the animal had become completely refractory to infection with the ordinary amount of the most virulent bacteria. Infection of susceptible animals showed that there was at first an increased alkalinity, but that this very soon subsided much below the normal, and remained so till death. It is probable, then, that the germicidal bodies of serum are only soluble or can only act in an alkaline medium.

Thus it has been shown that the liquids of an exudative inflammation have a manifold action. As a great part of the elements concerned in the combat with bacteria come from the vessels, the importance of the latter's share in the process is manifest. There is an experiment which emphasizes this fact. If the common femoral vessels in one leg of a rabbit are ligated and the leg below the point of ligation inoculated with pus cocci, a violent phlegmon and possibly gangrene results. Inoculation of an uninjured rabbit with a similar amount of the same culture produces only a localized abscess.

The fluid part of the exudate, then, flushes out the sewers,—it carries off detritus,—it supplies extra nourishment to the cells designated for repair and protects the system from invasion. The endothelial cells have some selective power on what passes by them. They remove or add certain substances to the plasma, and in addition have phagocytic powers. They can put forth processes and seize and incorporate bacteria, which, if they have escaped the emigrated leucocytes, are thus hindered from entering the general circulation.

The nervous system is intimately connected with all that occurs. But its action so far as known is as mysterious as ever. Beyond a few facts there is nothing definite. Histology has demonstrated that each cell of the heart has its particular nerve-filament, and as the heart is but a dilated vessel, it is presumable that each endothelial cell has its filament. Thus, with the aid of the central and peripheral vasomotor centres, the co-operation of the vessels in every inflammatory process is assured.

That the central nervous system does control to a certain extent the progress of events during a bacterial infection is shown by the following experiment.

Divide the vasoconstrictor nerve in one ear and the vasodilator in the other ear of the same rabbit. Then inoculate both ears with the erysipelas coccus. The ear in which the constrictor nerve has been cut rapidly recovers, while the other undergoes gangrene and perishes. If, now, in a fresh rabbit all connection with the central nervous system is severed by cutting all the peripheral nerves of the ears, and the ears are then similarly inoculated, the inflammation still appears and runs its course. Hence the central nervous system is merely an adjuvant in the various phenomena. It is simply a modifier of the process, important but not essential.

The same may be said of the temperature which accompanies an infection. Nothing very definite is known about it.

I have thus attempted to show that an inflammation is nature's response to an injury; that anatomically the difference between the reaction to a simple traumatism and the reaction to bacteria and their poisons is more one of degree than of kind. It is all an attempt to overcome a disaster, and the greater the disaster the more workmen—the more reparative elements—are required. When there is much *débris* to be cleared away a greater amount of labor has to be performed, and the parts concerned show great activity. In a simple incised wound there is but little destruction of tissue, and a few wandering cells are scavengers enough. A slight congestion of the vessels supplies most of them and enables the fixed cells to repair the damage. A large contused wound requires greater activity.

The solvent power of the cells is exerted upon the necrotic matter of an aseptic wound as upon bacteria and their products. This is manifested clinically by the aseptic wound fever, as it is called. It is the manifestation of the absorption of the ferments generated in the solution and removal of the dead matter in an aseptic wound. Micro-organisms provoke the same general response to the injury which they occasion. There is the congestion and swelling from the dilatation of the vessels and the exudation of plasma. With this goes the emigration of the leucocytes. There is always an initial negative chemiotaxis, although this may be of so short duration as to be scarcely perceptible. It is probably to allow the bactericidal substances, the fluid part of the blood, to at least weaken the bacteria. I speak of it as though there were conscious effort in the cellular elements, but I mean it in an evolutionary sense. It is a derivation of ages of cellular growth, combat, and development. Some of the wandering cells first approach and excrete a ferment. This may or may not be the same as that secreted by the phagocytes, which follow and absorb and digest both living and dead particles. The bactericidal action of the unformed elements and phagocytosis are two processes which go hand in hand; there is much doubt about the relative importance of each. But the general consensus of opinion seems to place phagocytosis in the secondary position.

This property of phagocytosis is possessed by the amœboid leucocytes of the blood and lymph, by many fixed cells, such as the endothelial cells of the vessels and serous membranes, by the epithelial cells of the intestine, which can ingest the cholera vibrio, and by the cells of the splenic pulp.

As soon as the destructive agency permits, repair by proliferation of the connective-tissue cells begins and proceeds as in an aseptic wound. Over all, with the mysterious general vitality which seems to be centred in it, is the guiding influence of the central nervous system.

